# The aging heart: exploring the role of Klotho in cardiac health/function

**DOI:** 10.20517/jca.2024.16

**Published:** 2024-12-19

**Authors:** Nastaran Daneshgar, Dao-Fu Dai, Chad E. Grueter

**Affiliations:** 1 Department of Pathology, University of Iowa, Iowa City, IA 52242, USA.; 2 Department of Pathology, Johns Hopkins University School of Medicine, Baltimore, MD 21287, USA.; 3 Abboud Cardiovascular Research Center, Department of Internal Medicine, Division of Cardiovascular Medicine, University of Iowa, Iowa City, IA 52242, USA.; 4 Fraternal Order of Eagles Diabetes Research Center, University of Iowa, Iowa City, IA 52242, USA.

**Keywords:** Klotho, cardiac aging, senescence, inflammation, heart failure

## Abstract

This review discusses the pathophysiological changes associated with cardiac aging and the potential therapeutic role of the anti-aging protein Klotho. It highlights key contributors to heart failure, such as arterial stiffening, myocardial fibrosis, and impaired cardiac relaxation, all of which lead to the declining function of the aging heart. This review also explores the regulation of Klotho expression, its various forms, and its impact on cardiac health, emphasizing its protective roles against oxidative stress, inflammation, and cardiac remodeling. Klotho’s potential as a therapeutic target for mitigating cardiac aging and improving cardiovascular health in the elderly is a central theme, making it a promising candidate for future interventions aimed at enhancing cardiac function and longevity.

## INTRODUCTION

**Figure 1.**
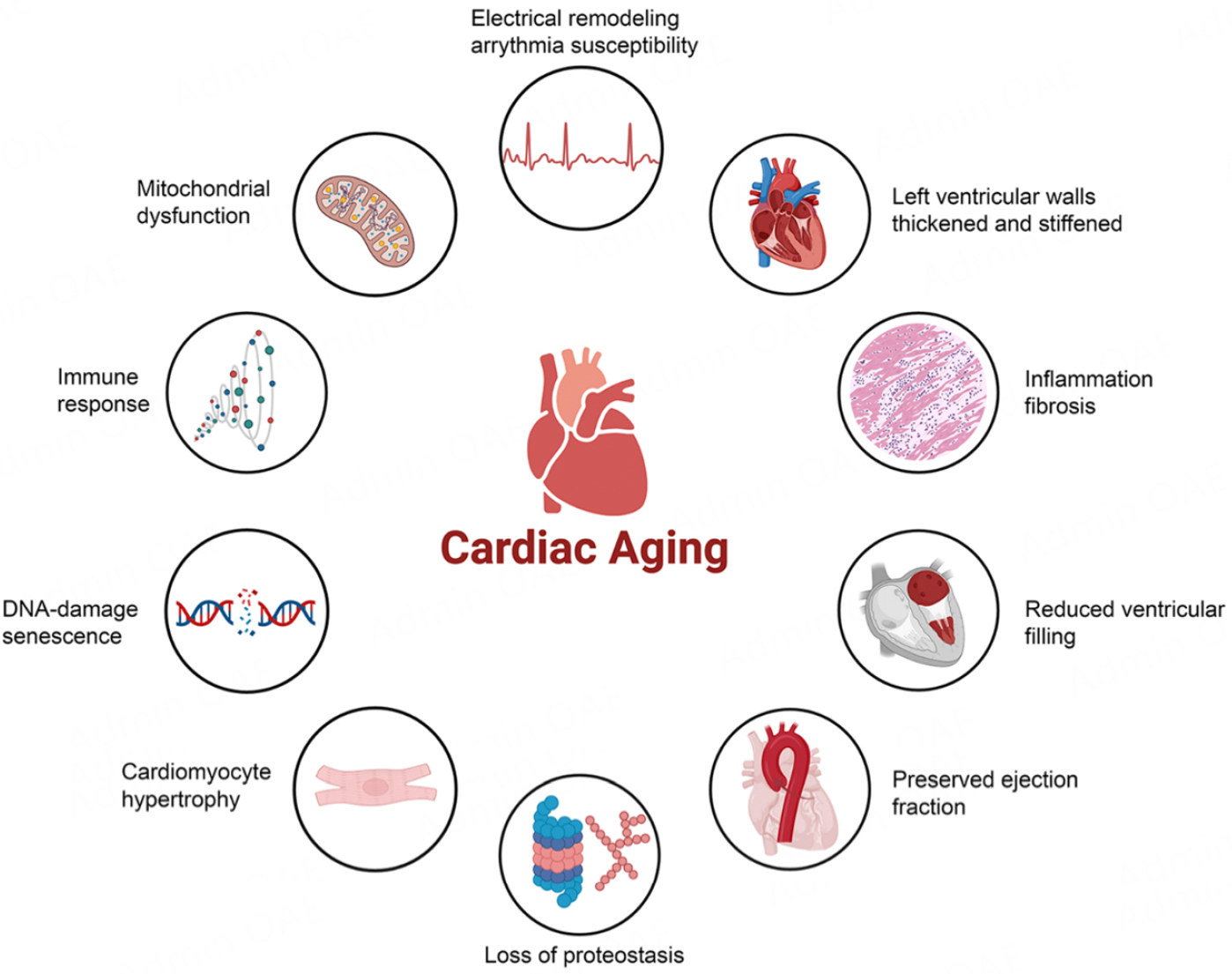
Cardiac aging hallmarks. This illustration highlights the pathophysiological changes associated with cardiac aging.

Cardiovascular disease (CVD) is the leading cause of noncommunicable disease deaths worldwide^[1]^. Heart failure (HF) prevalence progressively rises with each decade of life, with a significantly higher prevalence (up to 4-fold higher) among U.S. adults older than 65 years compared to those younger than 65^[2]^. As a result, HF is the leading cause of death in the elderly^[3]^. The risk of CVD increases with age due to several factors such as stiffening of arteries, changes in the heart muscle, and the accumulation of lifestyle-related risk factors such as poor diet and low physical activity levels^[4,5]^. The heart and blood vessels undergo many changes as a result of natural aging. These changes include cardiac hypertrophy, impaired relaxation of the heart during filling or diastolic dysfunction, the formation of excess fibrous tissue in the heart muscle or myocardial fibrosis, arterial stiffness, and endothelial dysfunction^[6–8]^ [Figure 1]. The most significant functional alteration observed in the aging heart is the gradual decrease in cardiac reserve^[9]^, which is also a crucial pathological characteristic of heart failure with preserved ejection fraction, the most prevalent form of heart failure in older individuals^[10,11]^. The existing cardiac magnetic resonance investigations suggest that the process of aging has an impact on the energy dynamics of blood flow in the left and right ventricles^[12]^, with a significant decrease in myocardial T1 values. These values, which measure the longitudinal relaxation time of the heart, suggest shorter relaxation times as hearts age. This reduction may indicate changes in the heart’s tissue composition, such as increased fat content and fibrosis^[13]^. The evidence presented establishes aging itself as a significant risk factor for the development of HF during the natural aging process. Therefore, targeting age-related pathways to counteract or reverse these age-related changes could ameliorate HF phenotypes in the elderly. One of the emerging anti-aging modulators is Klotho, and Klotho supplementation has been shown to mitigate age-related changes in various organs, such as the kidneys and brain. In this review, we thematically focus on recent advances in understanding the pathophysiological and molecular changes in cardiac aging and the potential anti-aging therapeutic implications of Klotho.

### PRODUCTION, STRUCTURE, AND REGULATION OF KLOTHO

Klotho has critical roles in aging, phosphate metabolism, and various cellular processes. It functions both as a membrane-bound co-receptor for fibroblast growth factor 23 (FGF23) and as a soluble protein with systemic effects. Through its interaction with FGF receptors, Klotho modulates phosphate and vitamin D metabolism, while its circulating form influences broader physiological processes. Structurally, Klotho contains two glycoside hydrolase domains, KL1 and KL2, which enable it to modify glycosylation patterns on target proteins, thereby modulating their function.

Klotho expression is dynamically regulated in normal physiological conditions and disease states. Regulation occurs at multiple levels, starting from gene expression driven by promoter activity and extending through various layers of epigenetic modulation and additional regulatory mechanisms. These complex interactions determine the overall expression levels of Klotho, influencing aging and other related processes.

Klotho is primarily expressed in the kidneys and brain, with some expression in the pituitary gland, pancreas, placenta, skeletal muscle, bladder, testis, aorta, ovary, colon, skin, and thyroid gland^[14–16]^. A notable decrease in Klotho mRNA and protein expression is observed in the majority of renal diseases, underscoring its sensitivity to pathological changes in the kidney^[17]^.

Klotho expression is significantly affected by a variety of conditions such as stress, hypertension, oxidative stress, diabetes, and various inflammatory disorders^[18]^. Additionally, Klotho gene expression is regulated by several transcription factors that bind to the promoter region. These include factors like E-box, Ap-2, PAX4, Sp1, and Oct-1^[19]^. Positive regulators such as PAX4, PPAR-γ, and vitamin D enhance the transcription of the Klotho gene. Conversely, NF-κB and angiotensin II have an inhibitory effect^[20,21]^. Interestingly, FGF23, which binds to the FGF receptor-Klotho complex, exerts a significant inhibitory effect on Klotho transcription^[22]^, explaining the observed reduction in Klotho levels in conditions such as chronic kidney disease that elevate FGF23 production^[23]^.

The complex regulation of Klotho at the genetic level allows for further control at the epigenetic level, which affects the availability and function of the protein. The Klotho promoter contains a high proportion of GC nucleotides, making it susceptible to DNA methylation^[24]^. In various malignancies, this can lead to gene silencing through DNA hypermethylation^[25]^. It has been shown that Klotho expression can be increased up to 3-fold in cells that typically do not express Klotho, through the use of DNA demethylating agents^[24]^. Chromatin immunoprecipitation assays have further linked the Klotho promoter region with H3K27me3, a marker that is highly upregulated in aging and contributes to epigenetic downregulation of Klotho with aging^[26]^.

Beyond these genetic and epigenetic factors, Klotho expression is also influenced by a range of physiological and environmental conditions that reflect the body’s complex response to internal and external stressors. Non-epigenetic factors contribute to the downregulation of Klotho in kidney disease. These include phosphate overload, vitamin D deficiency, hypoxia, angiotensin-II overactivity, uremic toxins, endoplasmic reticulum stress, ischemia-associated inflammation, hypomagnesemia, and albumin overload^[17]^.

The process of Klotho elimination from circulation has not been sufficiently explored. It has been shown that exogenous exogenous KL recombinant recombinant is present in the urine, which suggests the involvement of kidneys in its elimination^[27]^. Circulating Klotho is taken up by renal tubular epithelial cells in both proximal and distal tubules through unidirectional transepithelial transcytosis in a trafficking manner^[28]^. Therefore, kidney disease is expected to influence both the production and clearance of α-Klotho in a complex manner, highlighting the importance of further studies to elucidate these mechanisms. These insights could inform the development of therapeutic strategies targeting Klotho regulation and elimination pathways. Future research should focus on endogenous upregulators of Klotho and elucidate the mechanisms behind their regulatory effects.

### FORMS OF KLOTHO: TRANSMEMBRANE AND SOLUBLE

**Figure 2.**
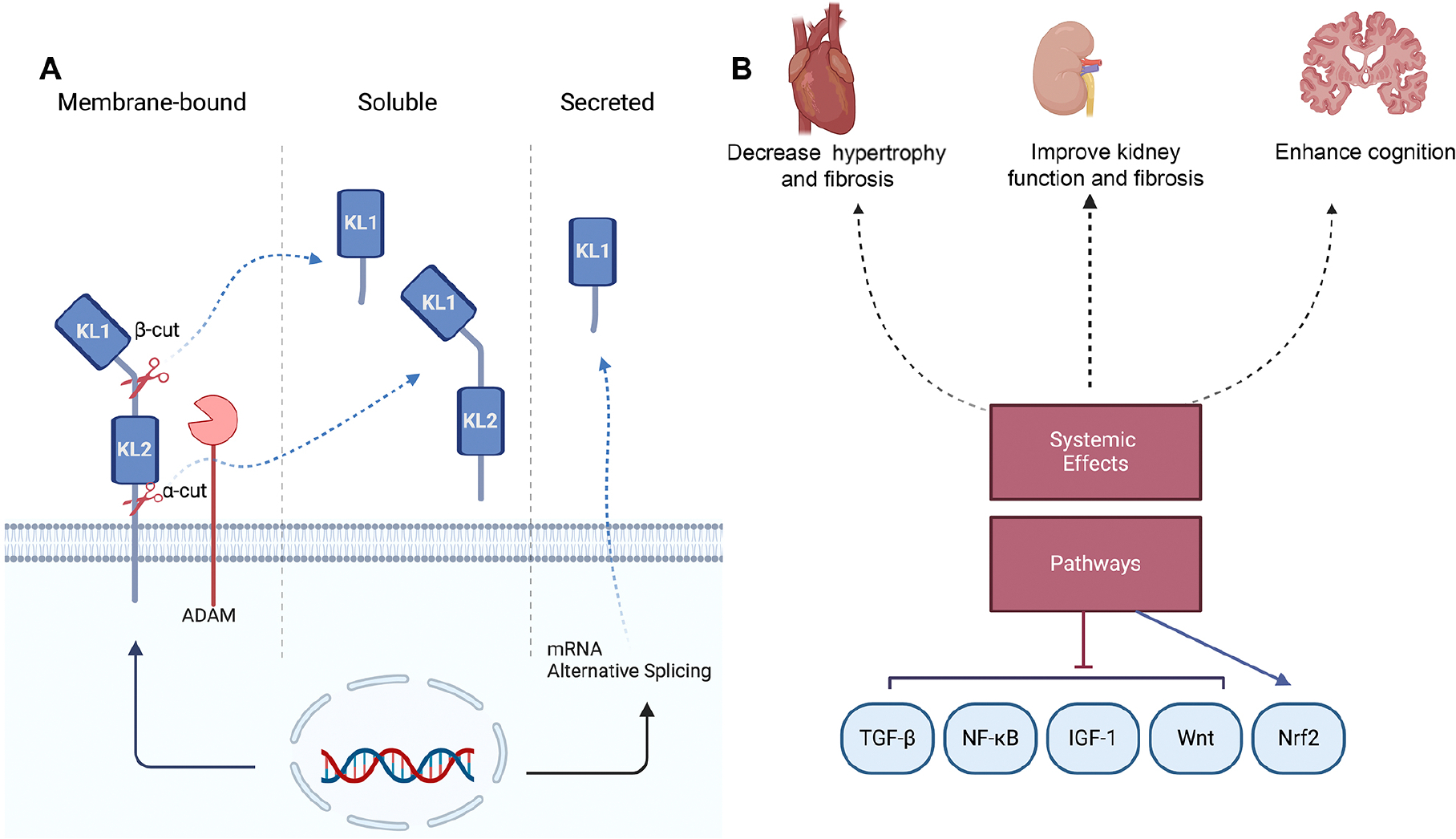
Klotho structure and multiple functions of Klotho. (A) Forms of Klotho protein: membrane-bound, soluble, and secreted. The membrane-bound form consists of KL1 and KL2. The extracellular domain can be cleaved by ADAM proteases, producing soluble Klotho. mRNA alternative splicing generates the secreted form of Klotho, which contains only the KL1 domain. (B) Systemic effects of Klotho on various organs and its role in reducing hypertrophy and fibrosis in the heart, improving kidney function, and enhancing cognition. Klotho exerts its effects through several pathways, including TGF-β, NF-κB, IGF-1, Wnt, and Nrf2.

The α-Klotho precursor protein exists in three forms: full-length transmembrane α-Klotho, shed or soluble α-Klotho, and secreted α-Klotho^[19]^. These forms play distinct and critical roles in various physiological and pathological processes influencing biological functions that impact renal health, metabolic processes, aging, and longevity. The full-length transmembrane α-Klotho consists of two distinct glycosyl hydrolase domains, KL1 and KL2, and is susceptible to cleavage by membrane-anchored proteases such as ADAM10 and ADAM17^[19,29]^. This cleavage results in the formation of soluble α-Klotho, which includes either KL1 (68 kDa) alone or both KL1 and KL2 (130 kDa) and is released from the cellular membrane into bodily fluids such as urine, blood, and cerebrospinal fluid (CSF) [Figure 2]. Soluble α-Klotho then functions as a hormone, regulating the activities of cells and tissues that do not produce Klotho^[30,31]^. Secreted α-Klotho, on the other hand, is produced through alternative splicing (65 kDa)^[32]^. Understanding the distinctions among these Klotho forms is crucial for their potential therapeutic applications in treating diseases associated with Klotho deficiencies.

### ANTI-AGING PROPERTIES

As an anti-aging protein, Klotho has been extensively studied for its potential therapeutic applications in aging. Klotho plays a crucial role in regulating oxidative stress and preventing age-related diseases such as diabetes, neurodegenerative disorders, and CVD^[33]^.

Klotho’s impact extends beyond merely combating diseases; it plays a crucial role in regulating kidney homeostasis through interactions with FGF23, a bone-derived hormone essential for maintaining the balance of calcium, phosphate, and vitamin D. Its effects on the renin-angiotensin system and other renal-related functions such as blood pressure, electrolyte balance, and hemodynamics emphasize its broad physiological implications^[34]^. Despite no specific receptor having been identified for soluble α-Klotho, it enhances the presence of transient receptor potential cation (TRPC) channel subfamily V member 5 (TRPV5) and renal outer medullary potassium channel (ROMK) 1 in the cell membrane. These channels are important regulators of calcium and potassium metabolism in the kidney^[35,36]^. Klotho regulates cell-surface glycoproteins and maintains calcium balance through its sialidase function, which involves removing α 2,6-sialylated LacNAc from theTRPV5 N-glycosylation branch to expose the galectin-1 binding site^[37]^. Additionally, Klotho regulates calcium flux entry and endothelial integrity by binding to vascular endothelial growth factor receptor-2 (VEGFR-2) and TRPC-1^[38]^. By enhancing these vital renal functions and promoting vascular health, Klotho’s actions contribute directly to mitigating the aging process.

Klotho’s influence is also evident in metabolic regulation. Klotho deficiency leads to significant metabolic effects, including decreased insulin production and increased insulin sensitivity, which collectively reduce energy storage and expenditure in Klotho knockout mice^[39]^. Klotho also downregulates NF-κB translocation and activation and transcription of downstream proinflammatory genes, preventing kidney damage^[40]^. Furthermore, Klotho regulates TGF-β1 signaling, which is closely related to kidney fibrosis. A newly discovered Klotho-derived peptide has been shown to preserve kidney function and ameliorate kidney fibrosis by binding to TGF-β receptor 2, disrupting the TGF-β/TβR2 interaction, and inactivating Smad2/3 and mitogen-activated protein kinases^[41]^. Recent studies have reported Klotho expression in podocytes of mouse and human kidneys, where it protects the glomerular filter through suppression of TRPC6-mediated Ca^2+^ influx by inhibiting phosphoinositide 3-kinase-dependent exocytosis of TRPC6^[42]^. However, Charrin *et al*., in a recent study, reported that Klotho is not highly expressed in podocytes, and podocyte-specific Klotho-deficient mice do not exhibit a glomerular phenotype or altered vulnerability to glomerular damage^[43]^. Hepatocyte-specific overexpression of Klotho, on the other hand, protected glomerular integrity and function by regulating the ER stress response^[43]^. These findings highlight Klotho’s central role in combating metabolic dysfunction and kidney damage, which are pivotal factors in the aging process.

Klotho’s renoprotective effects have been demonstrated in the prevention and treatment of ischemia-reperfusion injury-induced acute kidney injury (AKI) and in a chronic kidney disease (CKD) model induced by nephrectomy plus ischemia-reperfusion injury. Additionally, Klotho has been shown to prevent CKD progression and protect against cardiac remodeling in both models^[44]^.

In the brain, peripheral administration of soluble α-klotho enhances cognition and neural resilience in mice through the NMDAR-dependent pathway and induction of GluN2B cleavage^[45]^. Improved cognition following treatment with soluble Klotho has also been attributed to increased platelet factor 4 in plasma^[46]^. Intrahippocampal overexpression of secreted human α-Klotho improves object recognition, object location and passive avoidance memory, and overall memory formation in mice^[47]^. Klotho has also been studied as a therapeutic target for Alzheimer’s disease. In a mouse model of amyloid precursor protein/presenilin 1, treatment with Klotho for three months significantly reduced amyloid-β (Aβ) levels and improved cognitive dysfunction^[48]^. This effect is mediated through inhibition of NACHT, LRR, and PYD domain-containing protein 3 (NLRP3), promoting microglia M2 transformation and upregulation of Aβ transporter^[49]^. It has been shown that CSF Klotho levels are lower in individuals with Alzheimer’s disease compared to controls, lower in older adults compared to younger adults, and lower in females compared to males, suggesting a potential role of Klotho in brain aging and cognition^[50]^.

A recent report using data from 13,746 U.S. adults from the National Health and Nutrition Examination Survey (NHANES) confirmed low serum α-Klotho as an independent risk factor for all-cause and cardiovascular mortality in patients with hypertension, congestive heart failure, diabetes mellitus, and emphysema, and as an independent risk factor for all-cause mortality in those with renal insufficiency^[51]^.

Furthermore, serum Klotho levels were inversely and independently associated with metabolic syndrome prevalence, suggesting that higher Klotho levels could lower the risk of all-cause mortality in metabolic syndrome^[52]^. Several studies have reported higher soluble Klotho levels were associated with a lower risk of kidney function decline and incidence of CKD^[53]^.

Moreover, Klotho has been found to protect against hydrogen peroxide-induced injury in retinal pigment epithelial cells by upregulating Bcl-2 levels, downregulating cleaved-caspase-3 and Bax levels, therefore inhibiting apoptosis and promoting cell proliferation^[54]^. Taken together, the evidence shows the multifaceted benefits of Klotho in mitigating age-related diseases, enhancing cognitive function, and promoting renal and cardiac health. Klotho’s capacity to regulate oxidative stress, support metabolic health, and protect against cellular damage makes it a promising therapeutic agent for improving longevity and quality of life in aging populations.

### IMPACT ON CARDIAC HEALTH

The protective effects of Klotho extend to the cardiovascular system, illustrating its systemic benefits. Higher plasma Klotho concentrations are linked to a lower likelihood of having CVD^[55]^, highlighting its role in cardiac health. The mechanisms through which Klotho exerts its effects vary across different types of cardiac diseases, emphasizing its complex and multifaceted role.

#### Klotho and heart failure

Klotho deficiency has been strongly implicated in heart failure and its associated pathological processes. Klotho deficiency in Klotho hypomorphic [KL (−/−)] mice impaired heart function, inducing cardiac aging independently of phosphate metabolism^[56]^. Exogenous supplementation with secreted Klotho prevented heart failure, hypertrophy, and remodeling in KL (−/−) mice by inhibiting excessive cardiac oxidative stress, senescence, and apoptosis through suppression of glutathione reductase expression and activity in the heart via inhibition of transcription factor Nrf2. Additionally, cardiac-specific overexpression of GR prevented excessive oxidative stress, apoptosis, and heart failure in KL (−/−) mice^[56]^.

In models of isoproterenol (ISO)-induced cardiac stress, Klotho plays a significant cardioprotective role by mitigating hypertrophy, remodeling, and apoptosis. In Klotho-deficient mice, ISO-induced stress exaggerated pathological cardiac hypertrophy and remodeling. Conversely, Klotho overexpression in transgenic mice attenuated these effects by downregulating TRPC6 channels in the heart. Soluble Klotho inhibits TRPC6 currents in cardiomyocytes by blocking phosphoinositide-3-kinase-dependent exocytosis of TRPC6 channels^[57]^, further highlighting its role in protecting against stress-induced cardiac dysfunction. In addition to regulating ion channels, Klotho partially reversed isoproterenol-induced cardiac apoptosis in BALB/c mice by suppressing ER stress and downregulating activation of the p38 and JNK pathways^[58]^. Furthermore, Klotho reversed hypertrophic growth, disorganized myocardial fibers, and altered cardiomyocyte morphology in ISO-induced cardiac injury models, primarily by reducing oxidative stress. Notably, oxidative stress itself was found to suppress Klotho transcription, creating a potential feedback loop in ISO-induced cardiac pathology^[59]^. These findings collectively show Klotho’s diverse cardioprotective roles in mitigating stress-induced cardiac injury.

Investigation of the interaction between Klotho and the uremic toxin indoxyl sulfate (IS) in CKD-associated left ventricular hypertrophy (LVH) revealed a negative correlation between serum levels of IS and Klotho and both IS and Klotho levels were independently associated with LVH. Administration of IS in mice induced LVH and downregulated renal Klotho, with more severe LVH observed in Klotho-deficient mice. In vitro, Klotho inhibited IS-induced cardiomyocyte hypertrophy by reducing oxidative stress and blocking specific signaling pathways^[60]^.

These findings show Klotho’s role in preserving cardiac function by mitigating oxidative stress, hypertrophy, and remodeling, making it a promising therapeutic target for the prevention and treatment of heart failure.

#### Klotho and atherosclerosis

Klotho plays a significant role in mitigating atherosclerotic processes. Elevated serum FGF23 levels were positively correlated with increased carotid intima-media thickness (CIMT), a marker of atherosclerosis, while higher α-Klotho levels were inversely associated with CIMT. These findings suggest that α-Klotho may exert protective effects against the development of atherosclerosis in type 2 diabetes mellitus patients, potentially by modulating FGF23 activity and attenuating vascular calcification^[61]^.

Recent evidence further implicates reduced Klotho expression in the progression of atherosclerosis, particularly under diabetic conditions. A recent study demonstrated that Klotho expression was significantly decreased in smooth muscle cells in patients with diabetes and atherosclerosis. Consistently, ApoE-knockout mice with streptozotocin-induced diabetes on a high-fat diet exhibited lower Klotho expression in vascular smooth muscle cells, along with increased expression of TGF-β and MMP9 and enhanced phosphorylation of ERK and Akt. These findings suggest that reduced Klotho expression in vascular smooth muscle cells may exacerbate the development of atherosclerosis in diabetes^[62]^.

These findings show the critical role of Klotho in protecting against atherosclerosis, particularly in diabetic conditions, by regulating vascular homeostasis and preventing processes such as calcification, inflammation, and remodeling that contribute to disease progression.

#### Broader cardiovascular implications

Expanding upon the extensive cardioprotective effects discovered in several animal models, Klotho’s function goes beyond simply lowering oxidative stress and inhibiting hypertrophy. The complex functions of this include regulating important signaling pathways, such as the insulin/insulin-like growth factor-1 (IGF-1) signaling pathway, and activating forkhead transcription factors (FOXO), which in turn strengthen the body’s antioxidant defenses. Furthermore, the regulatory influence of Klotho on calcium and phosphate metabolism, achieved through interactions with TRPV5 and ROMK1 channels, highlights its overall significance in preserving cardiovascular health.

Klotho has also been linked to the prevention of CVDs through its regulatory effects on oxidative stress and insulin signaling. It suppresses insulin/ IGF-1 signaling, therefore activating FOXO and enhancing the expression of manganese superoxide dismutase. This, in turn, ameliorates oxidative stress by inhibiting the PI3K/AKT pathway, leading to the phosphorylation of FOXO3a and the regulation of mitochondrial antioxidant defenses^[63,64]^.

A recent study using a two-sample Mendelian randomization approach provided further insight into Klotho’s cardiovascular relevance. This meta-analysis revealed a suggestive inverse causal association between genetically predicted circulating α-Klotho levels and coronary artery disease, as well as a significant inverse association with atrial fibrillation. However, no causal associations were found between α-Klotho levels and heart failure, stroke, ischemic stroke or its subtypes^[65]^.

These findings collectively highlight Klotho’s crucial role in cardiovascular health, suggesting it is a promising therapeutic target for enhancing cardiovascular function against age-related decline.

### IMPACT ON STEM CELL REGULATION AND PROLIFERATION

Recent studies have highlighted the significant role of Klotho in regulating stem cell behavior and proliferation, offering insights into its potential therapeutic applications. Klotho regulates muscle growth and stem cell proliferation through Wnt signaling. Klotho binds to Wnt ligands, suppressing Wnt activity and downstream transduction^[66]^. This inhibition is particularly important in muscle stem cells, where Klotho acts as a natural inhibitor of canonical Wnt signaling^[67]^. Increased expression of Klotho initially leads to a temporary rise in satellite cell quantities and slows muscle fiber growth. This is followed by a phase of enhanced muscle growth, resulting in larger muscle fibers. Klotho’s effects are mediated by decreasing activity of the H3K27 demethylase Jmjd3 at the gene transcription level. This reduction in Jmjd3 activity increases H3K27 methylation, which in turn decreases the expression of genes involved in the canonical Wnt pathway, such as Wnt4, Wnt9a, and Wnt10a^[68]^.

Klotho enhances the proliferation, secretion, and migration abilities of bone marrow mesenchymal stem cells (BMSCs). Klotho-transfected BMSCs led to significantly improved renal function and reduced tissue damage in rhabdomyolysis-induced AKI mice models. The overexpression of Klotho increases protective factors like erythropoietin and IGF-1 while decreasing injury biomarkers^[69]^.

Klotho plays a crucial role in maintaining the hippocampal neurogenic niche. Klotho-deficient mice have reduced neural stem cell numbers, decreased proliferation, and impaired maturation of immature neurons. Conversely, Klotho overexpression increases neural stem cell numbers and proliferation, and enhances dendritic arborization of immature neurons, highlighting Klotho’s role as a regulator of postnatal neurogenesis^[70]^.

Klotho’s ability to regulate stem cell behavior and proliferation suggests its potential as a therapeutic target for mitigating age-related decline in the heart and other tissues. By modulating pathways such as Wnt signaling and enhancing the function of bone marrow mesenchymal and neural stem cells, Klotho not only promotes tissue regeneration but also protects against oxidative stress and cellular damage. These benefits make Klotho a promising candidate for developing interventions aimed at preserving cardiac function and overall health in aging populations.

### OXIDATIVE AND DNA DAMAGE

Mitochondria are the powerhouses of cardiomyocytes, playing a pivotal role in energy production essential for heart function. As the heart ages, mitochondrial function declines, significantly contributing to heart failure^[71]^. This decline stems primarily from the excessive production of reactive oxygen species (ROS) by mitochondria, which causes damage to mitochondrial DNA (mtDNA) and redox-sensitive proteins, thus exacerbating mitochondrial dysfunction and further ROS production. One of the major impacts of aging in the heart is the accumulation of damage to mtDNA. This damage is largely due to a lack of histones, reduced ability for DNA repair, and the proximity of mtDNA to the site of mitochondrial ROS (mtROS) generation^[72]^. Mitochondrial oxidation commonly produces 8-oxoguanine (8-oxoG), a marker of DNA damage caused by oxidative stress^[73]^.

Klotho plays a critical role in ameliorating oxidative damage and mitigating its downstream effects on DNA. Klotho has been shown to reduce isoproterenol-induced cardiac injury by specifically inhibiting ROS production, thereby protecting mitochondrial function and cardiomyocyte health^[59]^. However, oxidative stress can negatively regulate Klotho expression by suppressing the transcriptional activity of the Klotho gene promoter^[59]^. This dual relationship shows the importance of Klotho in maintaining redox homeostasis in the heart.

The DNA damage response pathway is also a key player in CVDs, with early studies correlating increased DNA damage with the severity of atherosclerotic disease^[74]^. Notably, the tumor suppressor BRCA1 is essential for protecting cardiac function by facilitating DNA repair and reducing apoptosis in cardiomyocytes. A loss of BRCA1 leads to poor cardiac function and increased mortality under stress^[75]^. Additionally, human atherosclerotic plaques in vascular smooth muscle cells exhibit a higher presence of accumulated double-strand breaks (DSBs) and activated ataxia telangiectasia mutated (ATM) compared to healthy tissues^[76]^. We have recently demonstrated that Klotho deficiency exacerbates DNA damage accumulation and diastolic dysfunction in the aged heart and soluble α-Klotho supplementation attenuated age-dependent DNA damage and improved cardiac diastolic dysfunction^[77]^. The activation of DNA damage response, as observed in Klotho deficiency, has been linked to more severe pressure overload-induced heart failure^[78]^. These findings emphasize the importance of genomic instability and DDR activation in controlling the progression of CVD.

Overall, the interplay between mitochondrial dysfunction, oxidative stress, and DNA damage, and the protective role of Klotho has significant implications for heart failure and other age-related cardiac conditions. Understanding these mechanisms opens avenues for targeted therapeutic interventions to improve cardiac health. Future studies are needed to elucidate Klotho’s specific involvement in the DNA damage response pathway, particularly in the heart.

### INFLAMMATION AND IMMUNE RESPONSE

Inflammation and fibrosis significantly contribute to the decline in heart function observed in the elderly. Chronic low-grade inflammation is a hallmark of aging, often referred to as “inflammaging”^[79]^. In the heart, this is characterized by the increased production of inflammatory cytokines and chemokines, which can lead to the activation of fibroblasts and subsequent fibrosis^[80]^. A recent study demonstrated that loss of the regulator of G-protein signaling 5 (RGS5) in pericytes triggers a pro-fibrotic reaction, marked by elevated gene expression of structural elements of the endothelial matrix (ECM) and pro-fibrotic growth factors TGFB2 and PDGFB^[81]^. Deletion of RGS5 leads to cardiac dysfunction, elevated collagen deposition, and fibrosis in the heart, which are characteristic signs of cardiac aging.

Klotho has been shown to have anti-inflammatory properties. Reduced expression of Klotho in diabetic mice leads to increased kidney inflammation^[82]^. Klotho deficiency, particularly when combined with a high-salt diet, causes higher blood pressure and infiltration of the kidneys by macrophages and T lymphocytes, exacerbating endothelial injury and inflammation in the kidneys^[83]^. Additionally, lower Klotho expression is seen in T-helper cells of elderly individuals and rheumatoid arthritis patients with joint inflammation, correlating with increased proinflammatory tumor necrosis factor-alpha (TNF-α) levels and associated renal fibrosis. Conversely, Klotho can suppress TNF-α expression and related inflammatory responses^[84]^. In a mouse model of cardiorenal syndrome induced by acute kidney injury, Klotho reduced inflammatory cytokines such as interleukins (IL)6 and 1β and TNF-α and provided cardioprotection by mitigating cardiac hypertrophy^[85]^.

Recent data suggest that microbiota play important roles in regulating the cardiac functions of aging hosts^[86]^. The cardiomyocytes in both aged humans and mice showed a higher presence of bacterial DNA. This was primarily due to gut microbial DNA, encapsulated within extracellular vesicles (mEVs), entering the bloodstream and subsequently infiltrating the cardiomyocytes of aging mice. Macrophages expressing Vsig4+ effectively inhibited the dissemination of gut mEVs, but the Vsig4+ cell population was significantly reduced in older animals. The administration of gut microbiota-derived extracellular vesicles (mEV) caused an inflammatory response in the heart and a decrease in the ability of the heart to contract in young mice lacking the Vsig4 gene. The removal of microbial DNA reduced the harmful effects of intestinal microbial extracellular vesicles (mEVs). Finally, replenishing the Vsig4+ macrophage population in older WT mice resulted in a decrease in the amount of microbial DNA and inflammation in the heart, while also enhancing heart contractility.

## CONCLUSION AND PERSPECTIVES

Cardiac aging is a complex and multifaceted process that significantly contributes to the prevalence of CVDs and HF in the elderly. As the global population ages, understanding the mechanisms underlying cardiac aging becomes increasingly crucial for developing effective interventions. Emerging as a candidate for combating aging, the anti-aging hormone Klotho is particularly noteworthy, both for its impact on cardiac aging and its broader anti-aging properties. Klotho plays a crucial role in reducing oxidative stress, inflammation, and pathological cardiac remodeling, all key contributors to cardiac aging and HF. Additionally, understanding the regulation of Klotho expression through genetic, epigenetic, and environmental factors offers potential therapeutic avenues for upregulating Klotho expression to combat cardiac aging and associated diseases. Furthermore, Klotho’s interaction with other pathways, such as SIRT1, emphasizes the complex network of molecular interactions involved in cardiac aging.

While Klotho has been extensively studied in contexts such as oxidative stress and inflammation, its potential involvement in cell cycle regulation, telomere maintenance, and DNA damage response pathways in the heart remains less studied. Future studies should aim to elucidate whether Klotho directly influences these mechanisms, as this could provide valuable insights into its role in mitigating cardiac aging and its associated pathologies.

Another important direction for future research is the identification and characterization of Klotho’s direct molecular targets and their behavior in specific stress models. While Klotho is known to modulate the activity of ion channels (e.g., TRPV5), signaling pathways (e.g., Wnt inhibition, FOXO activation), and metabolic regulators (e.g., FGF23), many of these interactions are inferred through indirect mechanisms or downstream effects. Advancing this area of research is essential to understanding Klotho’s precise molecular functions and their therapeutic potential.

Targeting Klotho for therapeutic purposes could involve strategies such as upregulating its expression, ass observed with both endurance and aerobic physical exercise^[87]^ or through pharmacological means^[88]^. Modulating the expression and activity levels of proteases such as ADAM10 and ADAM17 to enhance the production of soluble Klotho is not a feasible strategy to influence Klotho levels as these proteases also regulate the cleavage of numerous other protein substrates, raising concerns about potential off-target effects^[89,90]^. On the other hand, recombinant soluble Klotho delivery offers promising avenues for addressing Klotho deficiency in disease states such as chronic kidney disease and cardiovascular diseases.

In conclusion, Klotho represents a promising therapeutic target for addressing the challenges of cardiac aging. Continued research into its mechanisms and therapeutic applications could pave the way for novel strategies to enhance cardiac health and longevity, ultimately improving the quality of life for the aging population.

## Data Availability

Not applicable.
